# Measuring the Electronic Properties of DNA-Specific Schottky Diodes Towards Detecting and Identifying Basidiomycetes DNA

**DOI:** 10.1038/srep29879

**Published:** 2016-07-20

**Authors:** Vengadesh Periasamy, Nastaran Rizan, Hassan Maktuff Jaber Al-Ta’ii, Yee Shin Tan, Hairul Annuar Tajuddin, Mitsumasa Iwamoto

**Affiliations:** 1 Low Dimensional Materials Research Centre (LDMRC), Department of Physics, Faculty of Science, University of Malaya, 50603 Kuala Lumpur, Malaysia; 2 Mushroom Research Centre, Faculty of Science, University of Malaya, 50603 Kuala Lumpur, Malaysia; 3 Institute of Biological Sciences, Faculty of Science, University of Malaya, 50603 Kuala Lumpur, Malaysia; 4 Department of Chemistry, Faculty of Science, University of Malaya, 50603 Kuala Lumpur, Malaysia; 5 Department of Physical Electronics, Tokyo Institute of Technology, 2-12-1 O-okayama, Meguro-ku, Tokyo 152-8552, Japan

## Abstract

The discovery of semiconducting behavior of deoxyribonucleic acid (DNA) has resulted in a large number of literatures in the study of DNA electronics. Sequence-specific electronic response provides a platform towards understanding charge transfer mechanism and therefore the electronic properties of DNA. It is possible to utilize these characteristic properties to identify/detect DNA. In this current work, we demonstrate a novel method of DNA-based identification of basidiomycetes using current-voltage (I-V) profiles obtained from DNA-specific Schottky barrier diodes. Electronic properties such as ideality factor, barrier height, shunt resistance, series resistance, turn-on voltage, knee-voltage, breakdown voltage and breakdown current were calculated and used to quantify the identification process as compared to morphological and molecular characterization techniques. The use of these techniques is necessary in order to study biodiversity, but sometimes it can be misleading and unreliable and is not sufficiently useful for the identification of fungi genera. Many of these methods have failed when it comes to identification of closely related species of certain genus like *Pleurotus.* Our electronics profiles, both in the negative and positive bias regions were however found to be highly characteristic according to the base-pair sequences. We believe that this simple, low-cost and practical method could be useful towards identifying and detecting DNA in biotechnology and pathology.

Watson and Crick conceived the double helix structure of DNA in 1953^1^ using X-ray diffraction patterns obtained by Franklin Rosalind and Maurice Wilkins^2^. Despite slight modifications over the next few decades, features such as the hydrogen bond connected double helix structure remained valid to this day. Other features include the highly specific base pairing behaviors of Cytosine (C) with Guanines (G) and Adenine (A) with Thymine (T), which complements Chargaff’s rule^3^. Exposed outer edges of nitrogen in the bases meanwhile allows further hydrogen bonding with proteins, which play crucial roles in the replication and expression of DNA. As expected, these gives rise to various important biochemical and genetic processes^4^ that inevitably involve intrinsic charge transfer characteristics of the base sequences^5^. Based on previous studies^6^, DNA has been observed to be semiconducting when prepared at the right conditions, especially involving humidity and room temperature. It has also been shown that the rectifying response of each base pair to be characteristic of the base pair^7^,^8^ and base sequence^9^.

It is a known fact that metal-semiconductive junctions constitute a Schottky junction (Schottky diode), which imparts a rectifying effect on a current-voltage (I-V) profile as observed with a conventional p-n junction^10^. The same was observed with a DNA-metal (semiconductor-metal) junction, which actually constitutes a Schottky diode, and has been published and studied in numerous research papers^11^,^12^,^13^,^14^,^15^,^16^. Using conventional analysis technique, various electronic parameters such as ideality factor, barrier height and series resistance can be calculated from the I-V profiles^17^. This known aspect of DNA’s semiconductive behaviour as a Schottky junction and its characteristic I-V profile in complement with a metal contact is the basis for our own application in DNA detection and identification.

We utilized the properties of the DNA-Aluminium (Al) Schottky junction to respond variably in a highly characteristic manner in both the positive and negative bias regions towards different base sequences. These characteristic behaviors could be utilized in various applications especially in taxonomy, which is essential to biological science and is the basis of information exchange. Improved taxonomic awareness of edible mushrooms can help research on cultivation technology and breeding. For instance, an awareness of current taxonomy will assist to focus breeding strategy, avoiding crosses between two apparent ‘species’ (strains bearing different names) that are taxonomically identical. The plasticity of basidioma morphology of different species, especially those distributed in different regions of the world and the inaccurate identification of commercial isolates has led to numerous names for the same species and incidences of misidentification^18^. Mushroom belongs to the order of Basidiomycetes. Taxonomic identification of edible mushrooms based on morphological features can be unreliable and difficult. Furthermore, many species and strains cannot be grouped easily using morphological factors, because phenotypic variation in fungi can be affected by substrate and environmental factors^19^. The taxonomy of *Pleurotus* species, even with all efforts to illuminate it, has remained ambiguous^20^. According to Zervakis and Balis^21^, the taxonomic disagreements in the genus *Pleurotus* have risen for the following reasons; initial misidentification, absence of type specimens, instability of morphological characters due to environmental changes, limited reports on physiological characteristics, and lack of mating compatibility studies. Therefore, to illuminate the taxonomic position of species in the genus *Pleurotus* (earlier determined mainly by morphological features), many researchers started to classify these fungi also by molecular techniques^22^. However, the use of molecular methods to differentiate between closely related species are also difficult, because of the high similarity in conserved regions of closely related species, which may lead to misidentification^23^.

Our current method however involve acquiring electronic signature signals from semiconducting DNA molecules from different species of a closely related mushroom genus King Oyster: *Pleurotus eryngii* (KLU-M 1380), White Oyster: *Pleurotus floridanus* (KLU-M 1382), Gray Oyster: *Pleurotus pulmonarius* (KLU-M 1384), Seri Pagi: *Pleurotus giganteus* (KLU-M 1227), Wild *Pleurotus giganteus: Pleurotus giganteus* (KLU-M 1385), Abalone mushroom: *Pleurotus cystidiosus* (KLU-M 1388) and two control species from different genus Shiitake: *Lentinula edodes* (KLU-M 1386), Enoki: *Flammulina velutipes* (KLU-M 1387).

Primarily, this involves the fabrication of a metal-Semiconductor (DNA)-metal Schottky barrier diode, which quantitatively response to different DNA sequences when biased negative or positive. This variation in responses, which originates from DNA electronics were due to different base pair sequence giving rise to characteristic conductivity and current response with bias voltage. We observed characteristic I-V profiles for both the negative and positive bias voltages, from where various electronics parameters were measured. These quantified parameters were then used to effectively identify and detect the different mushrooms investigated in this work.

## Results

**Figure 1:**
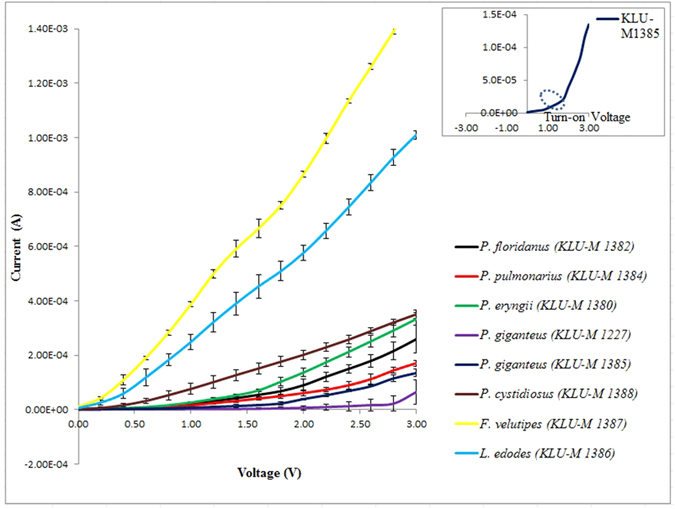
Positive biased I-V profiles for the six types of mushroom sub-species and two control species from different genus (*F. velutipes* and *L. edodes*). The insert illustrates the enlarged rectifying behavior of *P. giganteus* (KLU-M 1385) DNA, which shows a turn-on voltage of about 1 V, after which a clear magnification of current was observed. Each profile was averaged over six mushroom specimens.

**Figure 2:**
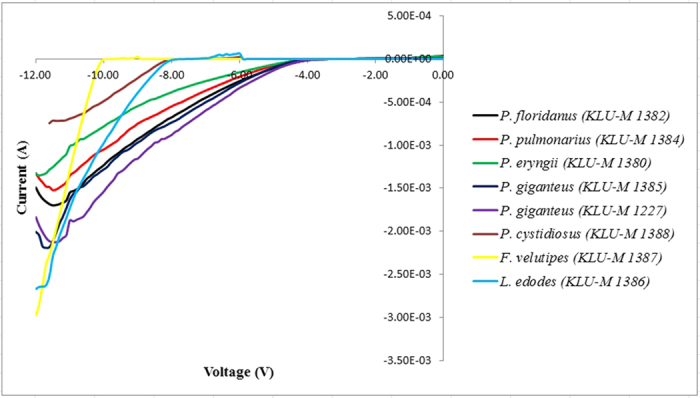
The negative biased (−12 to 0 V) I-V profile for the eigth types of mushroom sub-species, including the two control species from different genus *F. velutipes and L. edodes.* Each profile was averaged over six mushroom specimens.

Table 1 shows DNA purity and concentration using a NanoDrop spectrophotometer. Sample quality was assessed by the NanoDrop using absorbance measurements at 260 nm and 280 nm. The ratio of absorbance at 260 nm to 280 nm was provided to assess the purity of DNA. Pure DNA has an A260/A280 ratio of 1.8–2.0 and the A260/A230 ratio of 2.0–2.2^24^. However, a significant difference was observed when generating I-V profiles for each type of mushroom in both positive (Fig. 1) and negative (Fig. 2) regions. In the positive region, a clear rectifying profile can be observed for each mushroom. Figure 1 shows the comparison between different species of the same genus as well as two control species from different genus. It can be clearly seen from the profiles that the difference between the control species was much more significant compared to the ones generated by the different species of the same genus. A band of close yet distinguishable profiles was meanwhile observed for the mushroom specimens of the same genus in both positive (Fig. 1) and negative (Fig. 2) regions. This demonstrates the potential to utilize the I-V profiles to fingerprint and characterize the different species and genus. Both the figures also allows for measurement of various solid-state parameters for in-depth characterization of each type of DNA based on its base pair sequence electronics. Profiles generated from the positive bias region further enables the measurement of turn-on voltage, series resistance, shunt resistance, ideality factor and barrier height (Table 2). When the DNA-specific Schottky diodes were negatively biased, the characteristic profiles generated as shown in Fig. 2 can then be utilized to measure its knee-voltage, breakdown voltage and breakdown current (Table 3).

**Figure 3:**
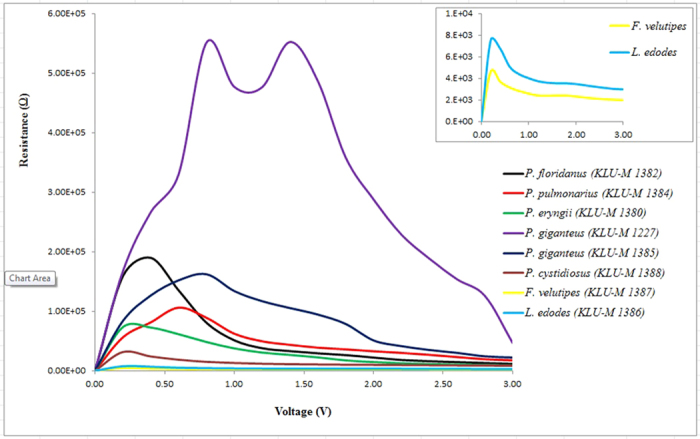
Resistance profile against bias voltage for all mushroom samples. Unlike the other species, *P. giganteus* (KLU-M 1227) however can be observed to generate two distinct maximums or shunt resistances, which may represent the characteristic charge transfer mechanism within its specific base pair sequence.

Table 2 lists the parameters calculated from the positive bias region. The results indicate characteristic values depending on the type of DNA used within the Schottky structure. Values of series resistance, R_S_ as shown in Fig. 3 were calculated from the junction resistance formula (R_S_ = 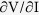) as measured from the I-V properties of the DNA diode. The figure generally demonstrates the series resistance values for regions 0 to 1 V, which can be utilized for identifying the DNA. Here, the maximum peak values were observed to occur characteristic to the type of mushroom (Table 2). Maximum value of series resistance belongs to *P. giganteus* (KLU-M 1227), while the lowest is for *F. velutipes (KLU-M 1387*). Region 1 to 3 V meanwhile demonstrates a decreasing trend in the series resistance complementing with the significant rectification of current beyond the turn-on voltage, which also show a predictable region. It was observed that the control specimens (*L. edodes* and *F. velutipes*) registered lower values of series resistance. These may be contributed in some ways to the evolving intrinsic dynamics of base-pair sequencing when introduced in the current environmental condition. Shunt resistance meanwhile was determined from the maximum resistance shown in Fig. 3 and its characteristic behavior can easily be deduced especially for the controls, which demonstrated the lowest values. Figure 3 clearly demonstrates this observation. The highest shunt resistance was observed for the *P. giganteus* (KLU-M 1227), while the lowest is for *F. velutipes (KLU-M 1387*).

**Figure 4:**
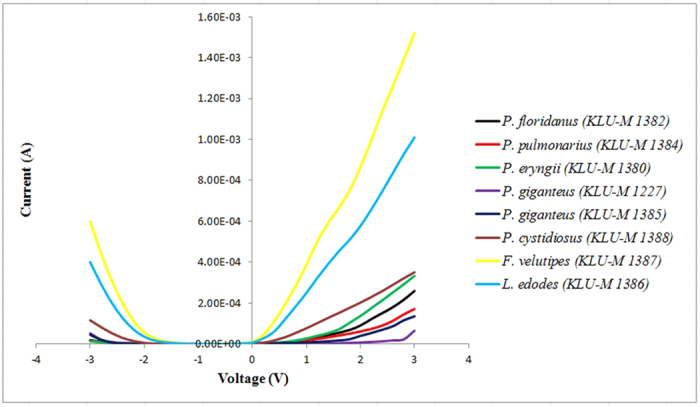
Semi-log I versus voltage profiles for the eight types of mushrooms.

**Figure 5:**
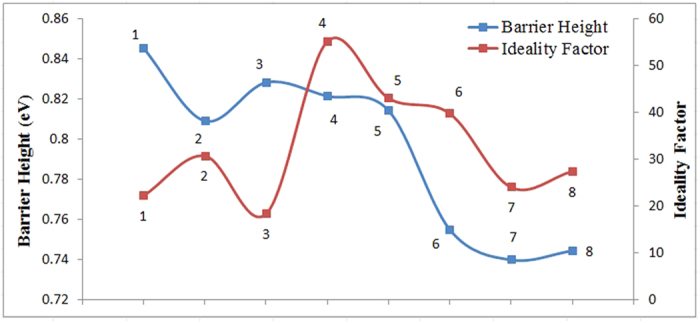
Ideality factor and barrier height profiles for all eight mushrooms; 1: *P. floridanus* (KLU-M 1382), 2: *P. pulmonarius* (KLU-M 1384), 3: *P. eryngii* (KLU-M 1380), 4: *P. giganteus* (KLU-M 1227), 5: *P. giganteus* (KLU-M 1385), 6: *P. cystidiosus* (KLU-M 1388), 7: *F. velutipes* (KLU-M 1387) and 8: *L. edodes* (KLU-M 1386).

Values of the barrier height of the DNA based Schottky diode meanwhile were calculated from the y-axis intercepts of the semi log-forward bias I-V plots (Fig. 4) using Equation (2). The barrier height is the connection potential barrier existing at the interface between the inorganic and organic layers, which in our case is the DNA/Al interface. Figure 5 shows the profiles plotted for the barrier height and ideality factor for the mushrooms based on the data listed in Table 2. The characteristic maximum obtained for the ideality factor for each mushroom therefore could be used as the identification method. Significantly similar values of the barrier height may indicate how close the mushroom species relates to each other, demonstrating that it may be classified within the same genus. Values obtained in this work vary from 0.74 to 0.85, which corresponds between the values generated by Al/n-Silicon (Si) (about 0.25 eV) and Al/p-Si (0.90 eV) Schottky junctions^25^. The closer the barrier height values are to zero, the more Ohmic the contacts are, indicating the importance of the choice of the metal contact. In this work, Al was utilized due to its easy availability and cheaper option.

Ideality factor representing the dominant charge transfer process in conventional diodes show a non-ideal condition in DNA based Schottky structures. Significant differences observed in these values may provide a detection or identification method, as sensitive ideality factor values are acceptably significant based on the profile shown in Fig. 5. However, some closer similarities are to be clearly observed within certain DNA sequences. We believe each parameter, including the ideality factor value represents certain properties pertaining to the dynamics of charge transfer within each respective base sequence, which may represent common features pertaining to the respective species investigated. As such, further interrogation on the genetics of these mushroom species may provide some key answers. The behavior remains constant and repetitions among the same batch samples generate highly reproducible patterns. The ideality factor determined from the slope of the linear region of the forward bias (ln (I)-V) characteristic through the relation in Equation (3) is a measure of conformity of diode to pure thermionic emission^26^,^27^. n equals to 1 for an ideal diode but here it demonstrates higher values. These high values can be attributed to the presence of interfacial thin film, a huge distribution of low Schottky barrier height (SBH) patches (or barrier inhomogeneity), rearrangement of electrons and holes in the depletion regions, and bias dependence of voltage of SBH^28^. The significant difference between the mushroom types may generally point towards the varying fundamentals including charge transfers and mobility underlying each DNA type according to its specific base sequences.

The DNA-specific negative biased profiles generated allows for discriminating knee-voltages and breakdown voltages that could also be applied as possible parameters for identifying different mushroom species. Similar values in the measured knee-voltage values may indicate the different species within the same genus or how closely related they are, while significantly higher values for the controls (−10.9 and −8.40) indicate different genus. Similar to a Zener diode, which operates in the reverse bias region in voltage regulating and switching applications, larger negative voltages or knee voltages are required to induce current amplifications. It was observed that similar breakdown voltages generated resulted in very different values of the breakdown current. There are many origins and sources of electrical breakdown, and “breakdown phenomenon” itself may not yet be a deterministic phenomenon. Statistical viewpoints may provide some discussions regarding the nature of the process. However, it should be noted that the electrical breakdown voltage is not merely an “injection” parameter. Still, these negative region parameters clearly demonstrate the possibility of utilizing them as a tool to identify different DNAs species.

## Discussion

The classification and taxonomic identification of species among the genus Pleurotus is difficult and unreliable because phenotypic variation can be effected by wide geographic ranges, geographic overlap of species, and continuing evolution and speciation^29^. Most taxa have different morphology from other taxa. Usually, the morphological differences within closely related species are much less than more distantly related ones. Some species look very similar, or maybe even outwardly identical, but are reproductively isolated. On the other hand, sometimes unrelated species obtain a similar look as a result of convergent evolution or even mimicry. In addition when relying solely on the morphological data, some species which were earlier determined to be different species would in fact turn out to be two distinct species. It would require further longer and more expensive DNA analysis to conclude that it is actually a single species. Our current method however involve directly acquiring characteristic or “fingerprinting” electronic signature signals from the respective semiconducting DNA sequence from different genus of a closely related species. Quantitative measurements such as (but not limited to) analysis of different parameters such as turn-on voltage, ideality factor, barrier height, series resistance, shunt resistance, knee-voltage and breakdown voltage can be utilized for rapid, simple and low-cost DNA/RNA identification.

Generally bulk properties of mushroom species are possible parameters available for identifying different mushroom species, for example, conductivity of mushrooms would be a possible parameter, however this is practically a hard task owing to the negligibly small differences among mushroom species. On the other hand, our focusing parameter is injection properties of mushroom species. As shown in Figs 1 and 2, and Tables 2 and 3, different mushroom species can provide clearly different parameter values that are available for the identification. This is the most important findings of our work. These methods of profiling can then be carried-out for trace diagnosis of DNAs, including pathogenic organisms and cancerous cells by establishing a database from where quick and rapid identification could be achieved. It should be noted that due to the ongoing process of evolution subjected to its corresponding environmental conditions, it is possible for slight variation in the electronic signal.

The initial results discussed in this current paper may demonstrate the possibility of utilizing DNA dependent Schottky barrier diodes as a kit to detect unknown DNAs by providing characteristic rectifying profiles and as an alternative way to discriminate closely related species. These could be then used to justify the importance of various solid-state parameters towards in-depth understanding of various fundamental physical, biological and biochemical phenomena in DNAs with different base pair sequences. Beside as DNA or RNA detection or identification tool, the DNA specific Schottky diodes may also be utilized as a tool for interrogating the underlying processes that could provide an insight into DNA’s damage and repair mechanism. As such, we have presented a novel method that provides significant quantitative data to clearly distinguish between different species and genus and between species in the same genus, in contrast to the limitations by the morphology based taxa.

## Methods

### DNA isolation

**Figure 6:**
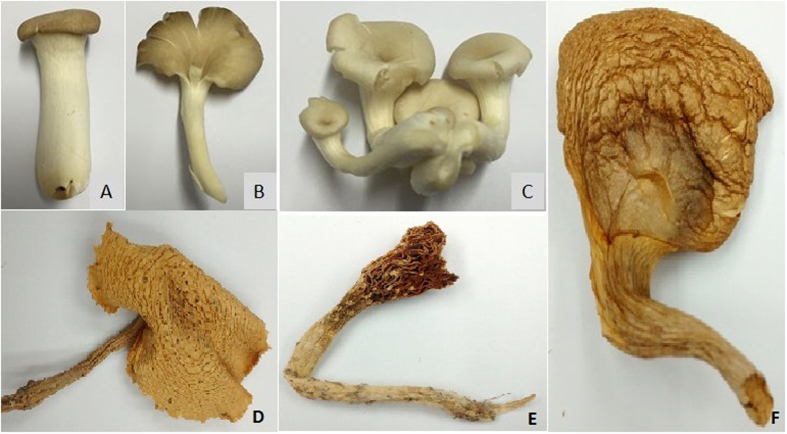
The six different species of edible mushrooms; (**A**) *Pleurotus eryngii* (KLU-M 1380), (**B**) *Pleurotus pulmonarius* (KLU-M 1384), (**C**) *Pleurotus floridanus* (KLU-M 1382), (**D**) *Pleurotus giganteus* (KLU-M 1227), (**E**) *Pleurotus giganteus* (KLU-M 1385) and (**F**) *Pleurotus cystidiosus* (KLU-M 1388).

Fresh fruiting bodies of six different species of edible mushrooms King Oyster: *Pleurotus eryngii* (KLU-M 1380), White Oyster: *Pleurotus floridanus* (KLU-M 1382), Gray Oyster: *Pleurotus pulmonarius* (KLU-M 1384), Seri Pagi: *Pleurotus giganteus* (KLU-M 1227), Wild *Pleurotus giganteus: Pleurotus giganteus* (KLU-M 1385), Abalone mushroom: *Pleurotus cystidiosus* (KLU-M 1388) and and two control species from different genus Shiitake: *Lentinula edodes* (KLU-M 1386), Enoki: *Flammulina velutipes* (KLU-M 1387), used in this study were purchased from local supermarkets (Fig. 6). Table 4 lists the Details of the six types of mushroom sub-species and two control species from different genus. Fresh fruit bodies of each species were dried using a food dehydrator. A small piece of dried tissue was cut and placed in a 2 ml micro centrifuge tube.

Total genomic DNA was extracted using Forensic DNA Extraction Kit (Omega Bio-tek) according to the manufacture’s protocol with modification. Initially, 100 μl STL buffer was added and samples were grinded for 2 min. Then another 100 μl STL buffer was added and incubated for 15 min at 55 °C. After brief centrifugation, 25 μl OB protease solution was added to the samples and mixed by vortexing and incubated for 45 min at 60 °C with occasional mixing. The samples were centrifuged to remove any droplets from the lids. Then 225 μl BL buffer was added to the tubes and incubated for 10 min at 60 °C. The tubes were briefly centrifuged and 225 μl absolute ethanol was added to the samples and mixed thoroughly by vortexing. After centrifugation, the aqueous phase was removed. Meanwhile, HiBind DNA columns were inserted into the collection tube. Equilibrium buffer (100 μl) was added into the columns and left for 4 min at room temperature. It was then spun at 13000 × g for 30 s by using microcentrifuge (Eppendorf MiniSpin plus centrifuge).

The entire sample including precipitation that formed was transferred from the microcentrifuge tubes to the earlier prepared HiBind DNA columns. HiBind DNA column containing samples were spun at 8000 × g for 1 min to bind the DNA to the matrix of columns. The collection tubes and flow through liquid were discarded. Columns were placed into new collection tubes. HB buffer (500 μl) was pipetted into columns and were centrifuged at 8000 × g for 1 min. The flow through liquid was discarded while collection tubes were reused. DNA were washed by pipetting 750 μl of wash buffer into column and centrifuged at 8000 × g for 1 min. Both flow through liquid and collection tubes were discarded. New collection tubes were used and DNA washed for second time by pipetting 750 μl of wash buffer into column and centrifuged at 8000 × g for 1 min. The flow through liquid was discarded while collection tubes were stored. The columns were centrifuged at 13000 × g for 2 min to dry the columns. All columns were transferred to new 1.5 ml Eppendorf tubes. Preheated elution buffer (50 μl) at 70 °C was added to columns and left for 3 min at room temperature. Columns were then centrifuged at 8000 × g for 1 min for elution. Second elution was done by adding another 50 μl of preheated elution buffer (70 °C) to columns and left for 3 min at room temperature. Columns were then centrifuged at 8000 × g for 1 min for elution. DNA samples contained in Eppendorf tubes were then stored at −20 °C freezer for future use. The DNA quality was finally measured using NanoDrop spectrophotometer.

### Fabrication of the Al/DNA Schottky barrier diode

ITO slides (KINTEC, Hong Kong) of width 1.1 mm, had a layer thickness of 100 nm with a dimension of 2 cm × 2 cm and 377.0 Ω/sq and about 10^4^ S/cm of sheet resistance and conductivity, respectively. The thickness of the Al wire electrodes used was (0.50 ± 0.05) mm and purity 99.999% (Sigma Aldrich, USA).

**Figure 7:**
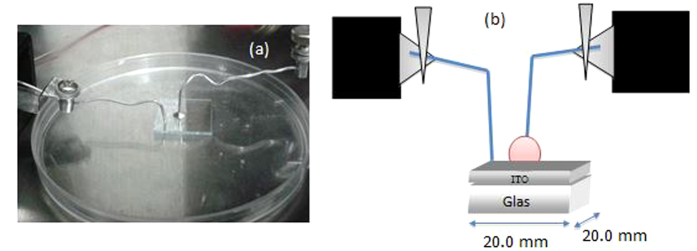
The prototype Al/DNA Schottky sensor used in this work (**a**) and its schematic diagram (**b**).

Prior to diode fabrication, the ITO substrates were thoroughly cleaned. To remove any surface contaminants, the ITO slides were rinsed with soap in an ultrasonic bath for 10–15 min. The slides were washed using deionized water and immersed in acetone and iso-polypropanol for 5 min each, followed by a final rinse using deionized water. Finally, the slides were dried using nitrogen gas to remove any water and other residues. 10 μl of DNA solution was then applied onto the cleaned ITO glass substrate. The entire preparation and fabrication process was undertaken in a Class 1 clean room to make sure the same environmental conditions (temperature at about 21 °C and humidity of 70 to 80 relative humidity (RH %) are maintained. Figure 7 demonstrates the schematic diagram and picture of the fabricated sensor for the measurement of the I-V characteristics (Keithley Electrometer, SMU 236).

### Acquisition of I-V characteristic profiles and calculation of electronic parameters

According to the thermionic emission theory, the I-V characteristic of a diode is given by^30^,^31^;


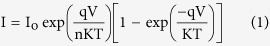



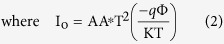


where q is the elementary charge, the applied voltage by V and effective Richardson constant by symbol A^*^ and equal to 1.3 × 10^5^ A/cm^2^K^2^ for ITO^31^,^32^. Symbol A meanwhile represents the active diode area, T the absolute temperature, K the Boltzmann constant, n the ideality factor of a SBD and Φ_bo_ the zero bias barrier height and R_S_ is the series resistance.

The saturation current I_0_ is determined by extrapolating the linear region of the forward-bias semi-log I-V curves to the zero applied voltage and 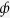 values are calculated from Equation (2) and can be written as Equation (3).


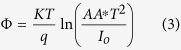


For values of V > 3kT/q, the ideality factor from Equation (1) can be re-written as:


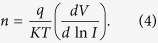


## Additional Information

**How to cite this article**: Periasamy, V. *et al*. Measuring the Electronic Properties of DNA-Specific Schottky Diodes Towards Detecting and Identifying Basidiomycetes DNA. *Sci. Rep.* **6**, 29879; doi: 10.1038/srep29879 (2016).

## Supplementary Material

Supplementary Information

## Figures and Tables

**Table 1:** NanoDrop result of the eight mushroom species investigated in this study.

**Sample**	***P. floridanus (KLU-M 1382)***	***P. pulmonarius (KLU-M 1384)***	***P. Eryngii (KLU-M 1380)***	***P. cystidiosus (KLU-M 1388)***	***P. giganteus (KLU-M 1227)***	***P. giganteus (KLU-M 1385)***	***F. velutipes (KLU-M 1387)***	***L. edodes (KLU-M 1386)***
Nucleic Acid Concentration ng/μL	53.9	53.1	54.8	53.1	53.7	54.2	54.4	53.3
A260/A280	1.95	1.85	1.98	1.83	1.80	1.85	1.86	1.81
A260/A230	2.21	2.19	2.24	2.23	2.22	2.18	2.20	2.19

The small variation in concentrations did not significantly influence the electronic properties of DNA-Al diodes [supplementary information: Figure S2].

**Table 2:** Electronic parameters (turn-on voltage, series resistance, barrier height, ideality factor and shunt resistance) calculated for all samples.

**Electronic Parameters**	***P. floridanus (KLU-M 1382)***	***P. pulmonarius (KLU-M 1384)***	***P. Eryngii (KLU-M 1380)***	***P. cystidiosus (KLU-M 1388)***	***P. giganteus (KLU-M 1227)***	***P. giganteus (KLU-M 1385)***	***F. velutipes (KLU-M 1387)***	***L. edodes (KLU-M 1386)***
Barrier height (eV)	0.8455	0.8092	0.8284	0.7550	0.8216	0.8144	0.7399	0.7445
Ideality Factor	22.24	30.78	18.38	39.81	55.22	43.08	24.04	27.49
Series Resistance (Ω)	11583.31	17545.23	9008.56	8562.30	46434.84	22256.29	1971.87	2970.79
Shunt Resistance (Ω)	189732.32	105983.35	73365.78	31525.69	552181.12	162134.77	4623.49	7567.92
Turn-on Voltage (V)	1.00	0.95	0.80	0.40	0.25	1.20	0.30	0.25

**Table 3:** Values of knee voltage, breakdown voltage and breakdown current for the negative region.

**Electronic Parameters**	***P. floridanus (KLU-M 1382)***	***P. pulmonarius (KLU-M 1384)***	***P. Eryngii (KLU-M 1380)***	***P. cystidiosus (KLU-M 1388)***	***P. giganteus (KLU-M 1227)***	***P. giganteus (KLU-M 1385)***	***F. velutipes (KLU-M 1387)***	***L. edodes (KLU-M 1386)***
Knee-voltage (V)	−4.00	−4.20	−4.20	−8.10	−4.10	−4.00	−10.9	−8.40
Breakdown voltage (V)	−11.40	−11.50	−11.90	−11.25	−11.30	−11.70	Not within the voltage range investigated	−11.80
Breakdown current x 10^−3^ (A)	−1.700	−1.523	−1.352	−0.716	−0.212	−0.0219	Not within the voltage range investigated	0.264

*F. velutipes* does not show the breakdown voltage within this region and were not measured since the range of investigation was only until −12 V.

**Table 4:** Details of the six types of mushroom sub-species and two control species from different genus.

**Common Name**	**Species Name**	**Specimen Code**	**Sources**
King Oyster	*Pleurotus eryngii*	KLU-M 1380	Imported from China
White Oyster	*Pleurotus floridanus*	KLU-M 1382	Locally grown (Damansara Sdn Bhd)
Gray Oyster	*Pleurotus pulmonarius*	KLU-M 1384	Locally grown (Damansara Sdn Bhd)
Seri Pagi	*Pleurotus giganteus*	KLU-M 1227	Origin from China
Wild *P. giganteus*	*Pleurotus giganteus*	KLU-M 1385	Collected from local forest
Abalone mushroom	*Pleurotus cystidiosus*	KLU-M 1388	Locally grown (Gano farm)
Shiitake	*Lentinula edodes*	KLU-M 1386	Origin from China
Enoki	*Flammulina velutipes*	KLU-M 1387	Korea
