# Supplementary material for: Measuring the Electronic Properties of DNA-Specific Schottky Diodes Towards Detecting and Identifying Basidiomycetes DNA
Source: Sci Rep. 2016 Jul 20;6:29879. doi: 10.1038/srep29879 (PMC4951751; doi:10.1038/srep29879)
Supplement: Supplementary Information [file srep29879-s1.pdf]

Measuring the Electronic Properties of DNA-Specific Schottky Diodes Towards Detecting and Identifying Basidiomycetes DNA

Vengadesh Periasamy[1*], Nastaran Rizan[1,2,3,4], Hassan Makttuf Jaber A-Ta'ii[1], Tan Yee Shin[2,3], Hairul Annuar Tajuddin[4] and Mitsumasa Iwamoto[5]

[1]Low Dimensional Materials Research Centre (LDMRC), Department of Physics, Faculty of Science, University of Malaya, 50603 Kuala Lumpur, Malaysia

[2]Mushroom Research Centre, Faculty of Science, University of Malaya, 50603 Kuala Lumpur, Malaysia

[3]Institute of Biological Sciences, Faculty of Science, University of Malaya, 50603 Kuala Lumpur, Malaysia

[4]Department of Chemistry, Faculty of Science, University of Malaya, 50603 Kuala Lumpur, Malaysia

[5]Department of Physical Electronics, Tokyo Institute of Technology, 2-12-1 O-okayama, Meguro-ku, Tokyo 152-8552, Japan

***Corresponding Author:** Associate Professor Dr. Vengadesh Periasamy, Low Dimensional Materials Research Centre (LDMRC), Department of Physics, Faculty of Science, University Malaya, Kuala Lumpur, Malaysia. Email: vengadeshp@um.edu.my, Tel: +60-379674038.

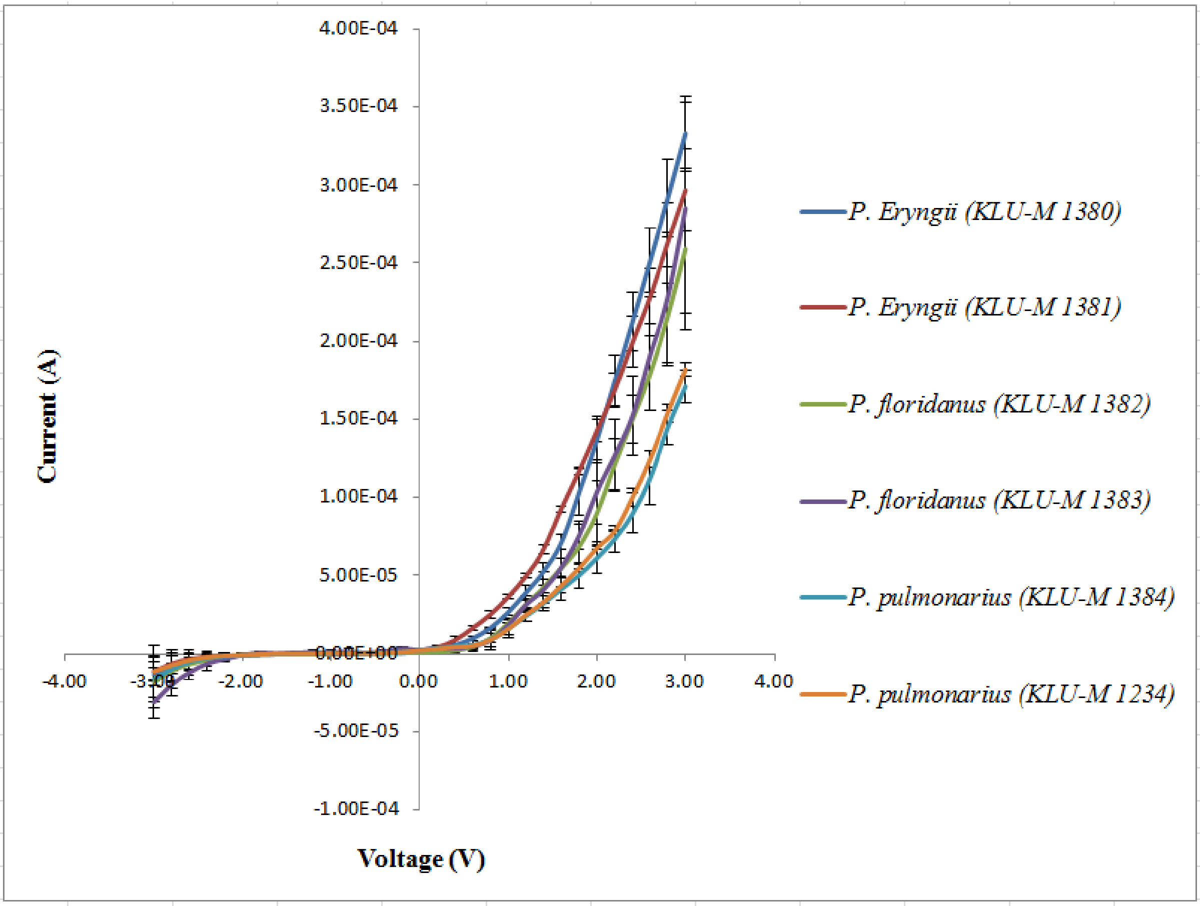

Figure S1: Comparison of *Pleurotus floridanus*, *Pleurotus pulmonarius* and *Pleurotus eryngii* I-V profiles measured in April 2015 and April 2016. *P. eryngii* (KLU-M 1380), *P. floridanus* (KLU-M 1382) and *P. pulmonarius* (KLU-M 1384) denotes samples measured in 2015, while *P. eryngii* (KLU-M 1381), *P. floridanus* (KLU-M 1383) and *P. pulmonarius* (KLU-M 1234) are samples from 2016.

Different Resources	n	ϕ	Rs	Shunt Resistance
P. floridanus (KLU-M 1382)	22.24	0.8455	11583.31	189732.32
P. floridanus (KLU-M 1383)	23.40	0.8378	10498.53	126190.93
P. pulmonarius (KLU-M 1384)	30.78	0.8092	17545.23	105983.35
P. pulmonarius (KLU-M 1234)	30.61	0.8154	16316.72	117895.18
P. eryngii (KLU-M 1380)	18.38	0.8284	9008.56	73365.78
P. eryngii (KLU-M 1381)	23.76	0.8050	10079.18	48657.07

Table S1: Electronic parameters between *P. floridanus*, *P. pulmonarius* and *P. eryngii* measured in April 2015 and April 2016.

The figure and table shown above illustrates the comparison between individual data from the same species carried-out in the positive region, but purchased from different resources measured at different times. Generally, the *P. pulmonarius* shows almost consistent readings for all the parameters investigated on April 2015 and 2016. This species, being a local species may indicate to being a well-adapted, stable and hardier commercial mushroom compared to the other Malaysian mushroom, which is the *P. floridanus*. In contrast, the species *P. eryngii* obtained from Thailand and China showed a slight variation, possibly demonstrating the continuous evolution and adaptation to the local Malaysian environment. As such, we believe our method is sensitive enough to pick-up the most subtle variations in base pair sequencing due to evolution, mutation etc.

Common Name	Species Name	Specimen Code	Sources
King Oyster	*Pleurotus eryngii*	KLU-M 1380	Imported from China
King Oyster	*Pleurotus eryngii*	KLU-M 1381	Imported from Thailand
White Oyster	*Pleurotus floridanus*	KLU-M 1382	Locally grown (Damansara Sdn Bhd)
White Oyster	*Pleurotus floridanus*	KLU-M 1383	Locally grown (Gano farm)
Gray Oyster	*Pleurotus pulmonarius*	KLU-M 1384	Locally grown (Damansara Sdn Bhd)
Gray Oyster	*Pleurotus pulmonarius*	KLU-M 1234	Locally grown (Gano farm)

Table S2**:** Details of same species, purchased from different resources.

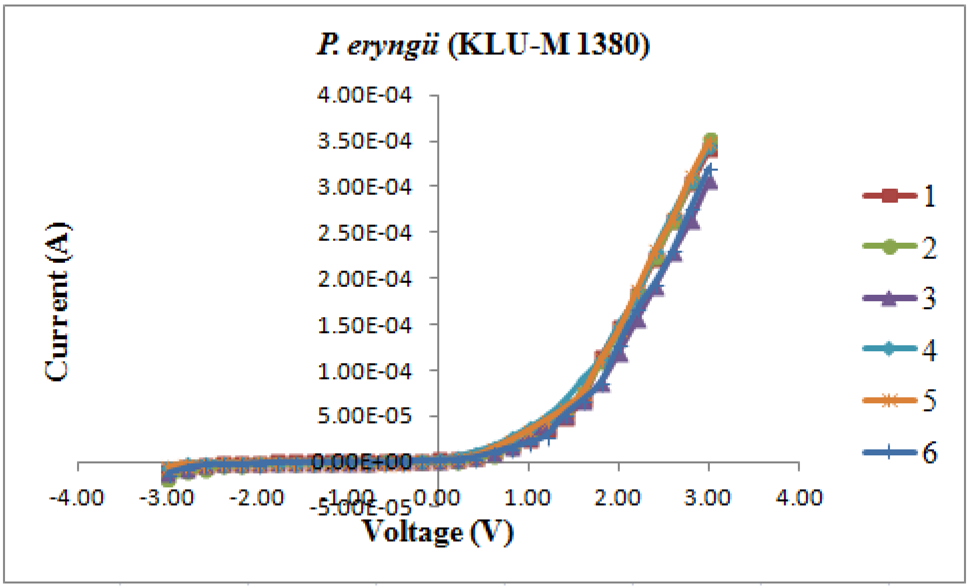

Figure S2: Different concentrations of DNA in King Oyster: 1=54.8 ng/uL, 2=54.2 ng/uL, 3=54.5 ng/uL, 4=53.8 ng/uL, 5=53.2 ng/uL, 6=53.1 ng/uL.

In this experiment, we extracted the same amount of fruit bodies of each species to obtain almost the same concentration of DNA for DNA extraction. Since NanoDrop is quite sensitive, there might be small variations with repeated measurements. It means that with a single sample, if read multiple times, it shows differences in the readings. The variation in the NanoDrop reading may occur due to the mixture being not homogenous and some DNA sedimentation. When the same sample is measured again, a new reading will be recorded depending upon the conditions of handling. Furthermore some factors such as calibration of NanoDrop before measuring the sample, air bubbles, dirty lens and its cleaning method between measurements, handling, pipeting, and solubility might allow some amount of variability in the results. It is understood that one cannot get exactly the same concentration of DNA as NanoDrop shows small variations in the reading.

The figure S2 is an example which clearly shows that the small variation in concentrations did not significantly influence the electronic properties of DNA-Al diodes. In other words, the

different electronic properties of DNA-Al diodes as greatly influenced by the variations in the base sequences rather than due to the different DNA concentrations.
